# Public support for restoration: Does including ecosystem services as a goal engage a different set of values and attitudes than biodiversity protection alone?

**DOI:** 10.1371/journal.pone.0245074

**Published:** 2021-01-19

**Authors:** Virginia Matzek, Kerrie A. Wilson

**Affiliations:** 1 Dept of Environmental Studies and Sciences, Santa Clara University, Santa Clara, CA, United States of America; 2 Queensland University of Technology, Brisbane, QLD, Australia; Universidad de Murcia, SPAIN

## Abstract

The ecosystem services concept has come into wide use in conservation and natural resource management, partly due to its appeal as an anthropocentric rationale for protecting and restoring nature. Proponents of the ecosystem services concept expect that presenting these arguments alongside biodiversity arguments should lead to a broader base of support for conservation. This raises the question of whether support for activities that ensure ecosystem service provision relates to different sets of core values, or environmental attitudes, than support for biodiversity protection. We surveyed adult Australians to evaluate the influence of values and attitudes on willingness to pay for different habitat restoration outcomes. We hypothesized that when restoration is framed with an anthropocentric rationale (such as ecosystem service provision), support for restoration would align more strongly with anthropocentric or self-centered values and attitudes. Specifically, we tested if preference for ecosystem service benefits over biodiversity attributes, as indicated by willingness to pay in different restoration scenarios, is more strongly associated with self-enhancing (Egoistic) than self-transcending (Altruistic and Biospheric) values, and more associated with a pro-use attitude towards nature (Utilization) than an anti-use attitude (Preservation). We found that support for habitat restoration is generally based on ecocentric values and attitudes, but that positive associations between pro-environmental behavior and Egoistic values emerge when emphasis is placed on ecosystem service outcomes. Individuals scoring higher on Egoistic/Utilization metrics were also more likely to anticipate disservices from restoration. Attitudes predicted behavioral intention (willingness to pay) better than core values. Our results support the notion that the ecosystem services concept garners nontraditional backers and broadens the appeal of ecological restoration.

## Introduction

Ecosystem services, the material and nonmaterial benefits that humans receive from the functioning of intact ecosystems, are increasingly seen as a justification for conservation action. In the field of restoration ecology, the notion that habitat restoration can return to society at least some of the natural capital that was lost when an ecosystem was degraded has gained wide currency [1–3]. Techniques to quantify and compare restoration’s impact on ecosystem services are now being used to set priorities [4, 5], evaluate projects [6, 7], or justify their expense [8, 9].

A driving force behind the adoption of the ecosystem services (ES) concept is the appeal of having an anthropocentric rationale for biological conservation, which may draw in new support from stakeholders unmoved by ecocentric or ethical arguments about preventing extinction or protecting untrammeled wilderness. Though the ES concept is undeniably controversial [10], proponents have argued that a “big-tent” or “pragmatic” approach to conservation, one that promotes environmental protection for reasons of human wellbeing alongside biodiversity, will result in more support for conservation [11–13].

Empirical studies testing whether ES arguments actually expand support for conservation are scarce. A study of projects undertaken by The Nature Conservancy found that ES-focused projects attracted more than four times as much funding, and from a wider variety of finance tools and corporate partnerships, than projects that had solely biodiversity goals [14]. With respect to ecological restoration, Clewell and Aronson [15] singled out a pragmatic approach based on preserving natural capital and mitigating climate change as the “most compelling” motivation for restoring ecosystems, and suggested that stakeholder appreciation of the economic benefits was necessary to generate broad public support for restoration.

The emergence of this anthropocentric rationale, and the increasing need for substantial human intervention in ecosystems to create and improve habitat, raise important questions about what moves people to support ecological restoration. There is a large body of literature suggesting that pro-environmental attitudes and behaviors depend most strongly on “self-transcending” core values such as altruism and concern for the biosphere [16–20]; others have shown pro-environmental attitudes and behaviors to be negatively associated with materialism [21] and social dominance [22]. This paper examines whether pro-environmental actions can appeal to those whose value orientation is more “self-enhancing” or who hold more instrumental or utilitarian views of nature [23] when ecosystem services are included alongside biodiversity.

Stern and others have segmented the core human values relating to environmental concern into Biospheric (nature-oriented), Altruistic (society-oriented), and Egoistic (self-oriented) strands [16, 19, 24]. According to the Value Belief Norm theory of pro-environmental behavior, any value orientation could potentially be associated with a pro-environmental action [25]. A person might sign a petition opposing pollution in streams because of a desire to swim in clean rivers (Egoistic–conscious of personal costs and benefits), to avoid harm to others (Altruistic–seeking to maximize social utility), or to keep nature pristine (Biospheric–accounting for the intrinsic value of nature). Though pro-environmental behaviors are most commonly associated with a Biospheric or Altruistic value orientation, we hypothesized that when restoration is framed with an anthropocentric rationale (such as ES provision), support for restoration would align more strongly with an Egoistic orientation.

We also investigated the role of environmental attitudes in structuring support for ecological restoration. According to cognitive hierarchy theory, people’s core values underlie their more malleable attitudes and beliefs, which in turn mediate the effect of their values on particular behaviors [26, 27]. We elicited respondents’ attitudes about the relationship between humans and nature with the Environmental Attitudes Inventory [28]. The EAI clusters people’s attitudes and beliefs along axes of Utilization and Preservation, analogous to ecological economists’ conception of “use” and “non-use” value. The Preservation axis is characterized by responses indicating enjoyment of nature, belief that nature is fragile, and ecocentric motivations for conservation. The Utilization axis engages belief in human dominance of nature, alteration of nature for societal ends, and anthropocentric motivations for conservation. We hypothesized that support for using ecosystem restoration to accomplish ES outcomes would depend more strongly on Utilization than Preservation attitudes.

In addition to its perceived benefits, restoration has potential to bring about significant disservices, or negative effects on wellbeing [29]. Restoration activities might increase ecological hazards or nuisances [30, 31], alter a community's "sense of place" [32, 33], or elicit negative feelings towards wildlife and wilderness, such as dislike, disgust, or fear [34, 35]. We therefore surveyed the public about potential drawbacks of restoration, hypothesizing that the more people exhibited Biospheric values and Preservationist attitudes, the less they would anticipate negative outcomes from restoration. We also expected that the Utilization axis of attitude, which tracks belief in human dominance over nature, would be associated with a high degree of concern about ecosystem disservices.

We also investigated how values and attitudes interact. While the Biospheric value orientation and Preservation axis would be expected to strongly overlap, and the Utilization axis and Egoistic value orientation should correlate, the relationship between an Altruistic value orientation and the two attitudes is difficult to predict. We anticipated that attitudes in general would be more closely related to behavioral intention than values [26, 27].

The pro-environmental behavioral intention we measured was willingness to pay (WTP), a concept from ecological economics. Because our goal was to generalize about preference for restoration outcomes in a national population rather than quantify the exact value of a proposed improvement at a specific place, we developed a generic restoration scenario, relevant to a nationwide audience, that showcased the delivery of multiple ecosystem services and biodiversity attributes. We also created an alternate scenario that promised only benefits to wildlife, and none to people, to see if the dependence of WTP on people’s values and attitudes varied between ES-focused outcomes and biodiversity-focused outcomes. This allowed us to contrast WTP as a relative, rather than absolute, measure of support for different restoration outcomes, avoiding some of the oft-cited reasons for skepticism about WTP as a measure of value (e.g., Venkatachalam, [36]).

The word “value” has multiple meanings in the social sciences [37]. The economic concept of WTP is a form of “assigned value,” the value imputed to a good by people’s preferences. Psychology is more concerned with “held values,” or the core, enduring principles at the center of an individual’s worldview. Studies that connect held values with assigned values are rare in the literature, but they are important to understanding conservation as a human endeavor [37, 38]. One reason for this is that social psychology can describe dimensions of motivation typically left unexamined by ecological economics, including moral and ethical motivations, and may explain why WTP often falls short of predicting actual behaviors [39]. In natural resource management, connecting values, norms, and beliefs to stakeholder preferences is critical to informed decision-making and the social acceptability of land management [40, 41].

It is a truism that many environmental issues and natural resource conflicts are, at bottom, clashes of values [42–44] and that those seeking to solve environmental problems ignore stakeholders’ values and attitudes at their peril [32, 41]. With this work, we wish to shed light on the “big-tent” view of environmental protection, which assumes that the ecosystem services rationale will invite in a new set of supporters with a different mindset. It is important to know if environmental campaigns based on appeals to self-interest or the promise of material benefits will be more or less compelling than moral or ethical arguments [45]. Clarifying the structure of environmental concern, and relating it directly to behavioral intention, should help improve environmental messaging and outreach to stakeholders (e.g., Bright et al. [46]).

## Methods

A large sample of adult Australians (n = 1,902) was recruited by a professional survey research firm and demographically matched to the Australian population on the basis of sex, age, state of residence, income, and education. The sample frame was the firm’s panel of 350,000+ Australian consumers with Internet access and respondents were recruited with small incentives (valued at ≤AUD$4), with quotas used to ensure demographic representation. Prior to the launch of the online survey, the questionnaire was pre-tested for length, intelligibility, wording, format, and question order among more than 25 respondents from six Australian states. Pre-test respondents were chosen to have no special knowledge of restoration, or concern for the environment, and were interviewed using verbal probing to understand their cognitive processes in reacting to the survey questions. Interviewees were also asked to suggest possible drawbacks of restoring woodlands in Australia, and we used the most commonly cited concerns to construct the question about ecosystem disservices.

To measure respondents’ values and attitudes, we used existing scales whose validity has been independently established, rather than inventing a new metric. For core values, we used the 12-item value scale of DeGroot and Steg [16], which scores responses on two self-transcendent value orientations (“Biospheric” and “Altruistic”) and on a self-enhancement value orientation (“Egoistic”) (S1 Appendix). For environmental attitudes, we used a short version of the EAI (Milfont & Duckitt 2010), with four statements on the pro-“preservation” axis and four statements on the pro-“utilization” axis (S2 Appendix).

We presented two restoration scenarios, a “biodiversity-only” scenario and a “biodiversity-plus-ecosystem-services” scenario (S3 Appendix). The biodiversity-only scenario (hereafter, BO) involved eradication of invasive species on an offshore island, and was written to preclude the possibility of humans benefiting directly from ecosystem services. The biodiversity-plus-ecosystem-services scenario (hereafter, BES) involved revegetation of a local woodland and the promise of a diverse set of potential ecosystem services and biodiversity improvements, including carbon sequestration, habitat, and recreation. Respondents viewed regionally specific versions of the scenario, using photographs and descriptions of local plant and animal species, to make each scenario more plausible as a potentially local restoration effort; otherwise, scenarios did not vary in wording or in the services promised. After viewing the BES scenario, respondents were asked to choose their preferred benefit from a list of those that could result from the restoration.

Half of the respondents saw the BO scenario first and the other half saw the BES scenario first. In both cases, respondents were told that private donations were needed to accomplish each restoration project and asked if they would be willing to donate. If so, respondents chose amounts from a payment card with exponentially increasing amounts, ranging from $1 to “more than $500” [47]. Those who were unwilling to pay (i.e., answered “no”) were asked to explain why, by choosing a set response or giving an open-ended explanation. Answers to this question were used to distinguish “true zeroes,” or people truly unwilling or unable to pay, from “protest noes,” those who objected to aspects of the payment scenario. When willingness to pay was treated as a binary category, only true zeroes were treated as “zero” and any specific dollar amounts were treated as “nonzero.” For analyses of WTP as a continuous variable, true zeroes were coded as $0 WTP, responses of “more than $500” were coded as $750, and protest noes were eliminated.

The scenarios devised to test whether the presence of an ecosystem services rationale increased WTP necessarily differed in other aspects, most importantly the location and type of restoration activity. We tolerated these differences because there are few plausible ecological restoration efforts that totally lack ES benefits (and none at all that would produce *exclusively* ES benefits, with no benefit to flora or fauna). The scenarios also necessarily differed in length and cognitive burden because we needed to explain only the biodiversity benefits in one scenario, but both biodiversity and ES benefits in the other.

We also gauged agreement with statements about the potential drawbacks of restoration (S3 Appendix), using a 5-point Likert scale from “strongly disagree” to “strongly agree.” Scores were combined into a Disservices Index by assigning the value 3 for “neither agree nor disagree,” 4 for “agree” and 5 for “strongly agree” with statements about potential disservices, and 2 for “disagree” and 1 for “strongly disagree” statements.

We used ordinary least squares regression models to understand the relationships between variables. To compare the impact of different attitudes and values on WTP and on the Disservices Index (DI), we used multiple regression and compared standardized partial regression coefficients among variables predicting WTP and DI. We used logistic regression to predict, from values and attitudes, the probability of making binary choices, e.g., preferring an ecosystem service over a biodiversity benefit. Biospheric and Altruistic value scores were square-transformed, and WTP amounts log-transformed, to reduce heteroskedasticity. All analyses were performed in R [48].

This research was performed according to standards designed to protect human subjects and approved by the Institutional Review Board of Santa Clara University, permit number 14-06-536, with Recognition University of Queensland HREC Approval 20140000747. Written consent was obtained from anonymous respondents by their acceptance of the consent form in the first page of the online survey.

## Results

In the BO scenario, Biospheric was the only significant value orientation; by contrast, in the BES scenario, the only significant value orientation was Egoistic (Table 1). An Altruistic value orientation did not significantly predict WTP in either case. If we do not adhere strictly to a standard of α = 0.05 for significance, we could note that all value orientations have p-values below 0.10 in the BO scenario; however, the Egoistic value orientation emerges as the only strong value predictor in the BES scenario. Regardless, in both scenarios, values were less influential than attitudes, as WTP was most strongly predicted by scores on the Preservation axis. The Utilization axis was negatively associated with WTP in the BES scenario. Values and attitudes explained relatively little of the variance in WTP: adjusted R^2^ values were 0.134 and 0.117 for the BES and BO scenarios, respectively.

**Table 1:** Regression statistics for the dependence of willingness to pay on values and attitudes in each restoration scenario.

		BO scenario^a^		BES scenario^b^		
	Standard β	*t*	*p*	Standard β	*t*	*p*
Altruistic	-0.063	-1.66	0.098	-0.027	-0.73	0.467
Biospheric	0.100	2.30	0.022	0.041	0.97	0.332
Egoistic	0.061	1.88	0.060	0.069	2.23	0.026
Preservationist	0.269	7.23	< .001	0.277	7.67	< .001
Utilizationist	-0.066	-1.67	0.095	-0.123	-3.30	0.001

^a^ Biodiversity Only scenario.

^b^ Biodiversity plus Ecosystem Services scenario.

We also asked how values and attitudes influenced respondents’ choice of an ES rather than a biodiversity attribute as the preferred outcome of restoration in the BES scenario (Table 2). The Egoistic value orientation and Utilization attitude were positively associated with choosing an ES attribute, while those more strongly expressing Preservationist attitudes were more likely to choose a biodiversity attribute.

**Table 2:** Logistic regression statistics for dependence on values and attitudes of choosing an ecosystem service instead of a biodiversity attribute as the preferred restoration outcome.

	Standard β	Z	*p*
Altruistic	0.061	0.439	0.661
Biospheric	-0.141	-0.893	0.372
Egoistic	0.335	2.88	0.004
Preservationist	-0.741	-5.66	<0.001
Utilizationist	1.117	8.11	<0.001

When respondents were asked to agree or disagree with two statements about the BES restoration—one that it would benefit people and the other that it would benefit nature—46% did not indicate equal agreement with the two statements. For these respondents, we tested the influence of values and attitudes on being more certain that the restoration would benefit nature more than people. This belief was associated with a higher Preservation axis score, a lower Utilization axis score, and a decreased Egoistic value orientation (Table 3).

**Table 3:** Logistic regression statistics for dependence on values and attitudes of belief that restoration will have greater benefits to nature than to people.

	Standard β	Z	*p*
Altruistic	1.252	1.764	0.078
Biospheric	1.458	1.643	0.100
Egoistic	-1.674	-2.611	0.009
Preservationist	1.353	2.031	.042
Utilizationist	-2.489	-3.647	<0.001

We found concern about potential undesirable outcomes from restoration, as expressed by the Disservices Index, decreased with more Preservationist attitudes and greater Altruistic value scores, and increased as respondents expressed more Utilizationist attitudes and Egoistic values (Table 4). The adjusted R^2^ was 0.34 for this analysis.

**Table 4:** Regression statistics for dependence on values and attitudes of the Disservices Index, a measure of concern that restoration will have important societal drawbacks.

	Standard β	Z	*p*
Altruistic	-0.051	-2.015	0.044
Biospheric	-0.002	-0.067	0.946
Egoistic	0.123	5.972	< .001
Preservationist	-0.109	-4.62	< .001
Utilizationist	0.465	19.11	< .001

Values and attitudes scores showed some degree of intercorrelation (Table 5). The strongest associations were between values and attitudes of the same type, i.e., there was a highly positive correlation between the two self-transcending values, Biospheric and Altruistic, and a highly negative correlation between the two environmental attitude axes, Preservation and Utilization. Altruism was positively related to Preservation and negatively related to Utilization. Biospheric values had the strongest relationship to attitudes, being positively correlated with Preservation and negatively correlated with Utilization. Egoistic values had the reverse relationship, but correlation coefficients were weak overall.

**Table 5:** Correlation coefficients^a^ for Pearson’s product-moment correlations between value orientations and attitude axes.

	Egoistic	Altruistic	Biospheric	Preservation	Utilization
Egoistic	1				
Altruistic	0.19	1			
Biospheric	0.18	0.66	1		
Preservation	-0.07	0.33	0.5	1	
Utilization	0.25	-0.25	-0.43	-0.54	1

^a^ All values are significant at p < .001.

We measured the internal consistency of the values and attitudes scores using Cronbach’s alpha. Higher values for this statistic mean that disparate items on the scale reliably measure the same value orientation or environmental attitude. For Egoistic, Altruistic, and Biospheric values, Cronbach’s alpha was measured at 0.76, 0.81, and 0.92, respectively. For attitudes, Cronbach’s alpha was 0.63 for Preservation and 0.70 for Utilization. A rule of thumb for sufficient consistency is a Cronbach’s alpha value of 0.7 or higher [49]. Reliability declines with fewer items on the scale, which may explain why (with only 4 items) the values are marginal for the two attitudinal metrics.

## Discussion

Our work produced three main findings. First, the results indicate a strong strain of ecocentrism in support for restoration, tempered by a lesser, secondary influence of egocentrism that appears when biodiversity protection and ecosystem service provision are explicitly contrasted. Though not unequivocal, these findings lend credence to our main hypothesis, that reliance on the anthropocentric rationale of ES provision can draw in support for conservation from those more concerned about personal gain and human benefit from the environment, and less responsive to ecocentric or biophilic arguments. Second, in agreement with our hypothesis, we found that altruism and desire for preservation predict people’s disinclination to believe that restoration can have significant drawbacks, while an egoistic/use-oriented orientation is associated with concern about potential drawbacks. Third, as predicted, environmental attitudes are generally more proximal to opinions or behavioral intentions expressed about restoration than are closely held, core values.

The most robust evidence for ES rationales tapping a nontraditional source of support for environmental action was when respondents were asked to choose a particular preferred benefit as the outcome of restoration. The selection of an ES outcome over a biodiversity attribute was positively associated with the Egoistic value orientation and the Utilizationist attitude cluster, and negatively associated with the Preservationist attitude. This suggests that those whose view of nature is that it should be exploited or managed for human benefit, and those who frame the costs and benefits of pro-environmental behavior in terms of personal gain, believe more strongly that restoration’s purpose should be to provide ecosystem services. This finding was echoed in responses to the general question about benefits to nature and benefits to humans resulting from the BES scenario restoration. The more a respondent tended toward Preservationist attitudes, and away from Egoistic or Utilizationist predilections, the less certain the respondent was that the restoration will have benefits to people commensurate with benefits to nature.

With respect to WTP as a measure of generic support for ecosystem restoration, the sole value orientation predicting WTP was Biospheric in the BO scenario, and Egoistic in the BES scenario. This again suggests that those whose value system is self-enhancing are more engaged by the prospect of restoration providing ES outcomes than of restoration solely aimed at biodiversity. However, it would be incorrect to say that support for restoration was not biophilic or ecocentric in the BES scenario, because the no-use Preservationist environmental attitude strongly predicting higher WTP in both scenarios, while the pro-use Utilizationist attitude negatively influenced WTP for BES. Also, the beta weights for Egoistic values in both scenarios are quite similar, even if the Egoistic value is not significant in the BO scenario.

Though many studies have related values and/or attitudes to respondents’ preference for environmental protection over a business-as-usual scenario, we know of only two that distinguish biodiversity protection and ecosystem service provision within a conservation scenario, as we did. Hicks and colleagues (2015) analyzed the values invoked by Pacific Island fishers for different ecosystem attributes and found that preferences were distributed across a diverse array of human values. Ecosystem characteristics associated with self-transcendent values included sanitation, protection from coastal hazards, and ecological function, while fishery goods and services were associated with self-enhancement, and were given the highest priority [50]. In another such study, attitudes among visitors to a California preserve influenced which ecosystem services and benefits they considered most valuable [51]. Two groups were identified from their responses to the New Environmental Paradigm (NEP) survey, strong biospheric and neutral NEP. The neutral NEP rated recreation higher, and biodiversity and scientific value lower, as components of value of an island preserve. This work, like ours, provides intriguing evidence that different assigned values for ecosystems engage different core values.

We can also make inferences relevant to our findings from psychometric studies that did not explicitly contrast biodiversity protection and ecosystem service provision, but instead compared particular management outcomes congruent with use and non-use value. For example, Swedish forest owners were surveyed about their attitudes on managing forests to emphasize preservation of ecological value, outdoor recreation, or timber production—land-use options which could be characterized as biodiversity protection, ecosystem service, and economic exploitation, respectively [52]. As might be expected, having self-transcendent values and a high NEP score were both positively associated with the ecological management attitude and negatively associated with the economic management attitude. But the ecosystem service attitude fell somewhere in the middle, with weaker dependence on NEP and no significant relationship with self-transcendent values. This is consistent with our finding that ES-focused management relies less heavily on an ecocentric worldview than does pure preservation.

Recreation as an ecosystem service was also the focus of a survey of winter visitors to Yellowstone National Park [53], in which people whose attitudes aligned with a highly biospheric axis (“naturalists”) had different opinions on park management issues from another group whose attitudes were characterized as “human-oriented.” Generally, naturalists favored management actions that would restrict human use of the park, while the human-oriented group was much more likely to desire expansion of recreation opportunities and amenities. Similarly, among Swedish respondents, the influence of norms and beliefs on support for conservation differed between wilder ecosystems with high ecological value and a cultivated town park valued mostly for recreation [54], with support for the town park being much more weakly associated with biospheric values and norms. For residents of Norway shown photos of various landscapes, wildland attractiveness was associated with the ecocentric value orientation, and farm attractiveness was associated with the anthropocentric value orientation [55]. In all of these studies, valuation of nature as a wilderness with high ecological value was more strongly aligned with a biospheric orientation than was valuation of nature as a place that humans enjoy.

Though not a contrast between biodiversity protection and ES provision per se, a result reported in Raymond and Kenter [56] is also relevant to our present findings. Rural land managers in Australia were surveyed about their intention to plant native vegetation on their farms, a scenario that entails many of the same ecosystem service benefits as our BES scenario. They found that no value orientations directly predicted planting behavior, but biospheric concern indirectly influenced behavior via beliefs and norms. This suggests that among people already invested economically in the exploitation of their land for ecosystem goods, restoration of non-use value, or ecosystem services, is more associated with Biospheric values than Altruistic or Egoistic values.

Together with our results, these findings collectively suggest that the commonly observed association between biospheric values and pro-preservation environmental attitudes applies best when the scenario considered by respondents is preservation of wild nature for the benefit of flora and fauna, with humans excluded. However, when intact ecosystems are anticipated to provide benefits that humans can enjoy, the biospheric orientation is less influential, and competing self-enhancing values or use-oriented attitudes can also explain people’s support for conservation. This lends support to the “big-tent” view that conservation efforts will benefit by drawing in support from nontraditional quarters with a more anthropocentric argument for habitat creation, restoration, and improvement.

An important caveat to this result is that it does not address the possibility that framing restoration outcomes in terms of ecosystem services actually results in less support for restoration by crowding out moral, ethical, or cultural motivations for conservation [57–59]. Using an ES rationale will not result in increased support for restoration if the additional contributions of those who express self-enhancing values and attitudes (and are newly attracted by the ES framing) are offset by the decreased contributions of individuals whose self-transcending values and attitudes are repelled by a focus on tangible human benefits. We did not design our study to directly answer this question, but two simple analyses from our dataset can shed some light on it. First, we can ask if the overall willingness to pay—the sum of all respondents’ answers to the WTP question—is lower in the BES scenario, which would suggest that overall outcomes for conservation were poorer under the ES frame. When we analyzed the data this way, we found that the sum of all respondents’ WTP was 20% higher in the BES scenario than the BO scenario. Second, we can ask if the BES scenario has higher rates of protest responses, which might occur if an ES framing was turning off more potential supporters than a biodiversity argument. In fact, we found that protest rates were significantly lower in the BES scenario. Therefore, we have no evidence that an ES rationale is crowding out traditional motivations for restoration support and resulting in poorer outcomes overall. Narloch and colleagues [60] found that crowding out was most likely to occur in response to a payment-for-ecosystem-services cheme where there was strong pre-existing community cultural expectation of conservation, but that “crowding in”—i.e., a “big tent” result—was possible where these conservation norms were initially weak. Our Australia-wide study, with its highly diverse and geographically dispersed participants, seems more likely to fit the latter scenario.

Another caution in interpreting our results is that the BES and BO scenarios differed in other respects besides the presence or absence of ecosystem services. For instance, we cannot rule out the possibility that people with higher Egocentric value orientations or Utilizationist attitudes responded more positively to the BES scenario because of its proximity, familiarity, or place significance. The BES scenario shown was specific to the respondent’s region, while the BO scenario occurred in a location vaguely described as “offshore.” Also, the BES scenario concerned a woodland habitat whose flora and fauna might be more familiar or desirable to the respondent than the seabird rookery described in the BO scenario. People have different affinities for different habitats and landscapes [61–64] and proximity to the benefit is known to influence people’s assessment of the value of an ecosystem service [65–68]. This does not necessarily invalidate the proposition that the subjects are responding to an anthropocentric rationale, because the effect of these scenario differences may be to enhance the respondents’ confidence that the proposed restoration yields a benefit that they understand, desire, and are close enough to receive. But we have no data that can tease apart an independent effect of the sense of place from the existence of the services. Because biodiversity enhancement and ecosystem service provision typically go hand in hand, it is difficult to formulate realistic scenarios that can separate the two without introducing different forms of bias, such as place significance.

We were surprised by the way Altruistic values explained behavioral intentions and attitudes in this study. Other work has frequently shown an influence of altruism on WTP (e.g., Ojea and Loureiro [69]. Although we expected that altruistic individuals would regard restoration as beneficial to society at least in the BES option, the Altruistic orientation had no association with WTP in either scenario. The Altruistic orientation also had no relationship with choosing an ES as a preferred outcome of restoration, nor with seeing a distinction between societal and biospheric benefits of restoration. Instead, and counterintuitively, Altruistic values appeared to make respondents less concerned about negative (societal) impacts of restoration disservices. It has been shown that Altruistic values and Biospheric values can be difficult to distinguish in relation to pro-environmental beliefs [19, 70], possibly because the Altruistic item set includes aspects of universalism that also strongly relate to a biospheric orientation. In our dataset, the Altruistic orientation was highly positively correlated with Biospheric orientation and the Preservation attitudinal axis, which may explain the generally low explanatory power of altruism throughout our analyses, as well as the result negatively associating Altruistic values with the Disservices Index.

Ecosystem disservices are poorly studied compared to services [71] and rarely considered simultaneously with benefits [72, 73]. We know of no other research relating core values and environmental attitudes to the perception of disservices, although a few studies have shown that these perceptions may differ cross-culturally [74], between rural and urban dwellers [75], across income and ethnic groups [76], with proximity to the disservice [77], and among people with different pro-environmental behaviors [78]. Given the dearth of work on this topic, we are unable to contextualize our finding that disservices were more concerning to people with Egoistic values and Utilizationist attitudes. It seems clear enough, given that the Utilization scale includes items about “remaking nature…when it is uncomfortable and inconvenient for humans” and conservation being placed in opposition to people’s standard of living, that high Utilization scores would be associated with perception of disservices. However, we found the lack of influence of the Biospheric value somewhat puzzling, in that people who believe nature has intrinsic value should be relatively unconcerned that ecosystems in their natural state have both negative and positive consequences for people. And more Altruistic people, whose values center on maximizing social good, were less likely to agree that restoration could have negative impacts on the economy, fire safety, and other societal issues. One reason for the lack of clarity might be that our DI lumps together disparate concerns that were identified in focus groups as likely concerns about woodland restoration; these concerns may not all engage the same sets of values and attitudes. As ES frameworks for conservation and public investment become more prevalent, we will need to further investigate what shapes the public’s perceptions of disservices.

Finally, we found that attitudes were generally more closely related to the behavioral intention of WTP than values, although the overall predictive power of all the psychometric measures was relatively small. Our finding that attitudes were more proximal to behavior is consistent with cognitive hierarchy theory [27], which states that (more specific, more numerous) attitudes mediate the effect of (more general, less numerous) values on environmental behaviors. For example, in another study that combined measures of both values and attitudes, Biospheric values had significant indirect, but not significant direct, influences on willingness to pay a premium for organic food [79], leading the authors to conclude that pro-environmental attitudes mediated the effect of Biospheric values.

One question about our approach is the appropriateness of using WTP as a measure of support for restoration. We chose WTP because restoration is an intervention that incurs costs and that brings about a change in environmental benefits to people and to habitat, making our scenarios both realistic and akin to a cost-benefit analysis. Because the relationship between values and actions is apt to be mediated by the cost of the action [80], we sought to make the costs both transparent and personal. But non-monetary metrics, such as measures of improved psychological wellbeing as a result of contact with nature, capture different aspects of assigned value [81, 82] and may relate differently to core values and attitudes. Studies of WTP are also plagued by protest responses, which must be discarded for the analysis and thus represent a segment of the public whose values go unexamined. Other researchers have measured assigned value by asking respondents to allocate a budget of value points instead of promising to pay money [83], which may avoid the protest response problem. We also note that we assume WTP accurately represents support for restoration, but that support could find other outlets besides a monetary donation (e.g., volunteering). We also implicitly assume that values and attitudes are stable, and affect environmental behavior like WTP, but we do not consider the possibility that framing conservation issues in anthropocentric or ES-focused terms has an effect on attitudes or even values—a matter of some debate in the literature, but outside the scope of this paper. Before we can be certain that the ES rationale is successful at garnering more support for conservation, we will need to have a deeper understanding of what that support entails, and a more diverse methodological approach to these questions.

## Supporting information

S1 Appendix Value orientations and survey items. (PDF)

S2 Appendix Attitude axes, scales, and survey items. Asterisk indicates reverse-coded item.
(PDF)

S3 Appendix Survey questions analyzed for this paper. (PDF)
