# Supplementary material for: Public support for restoration: Does including ecosystem services as a goal engage a different set of values and attitudes than biodiversity protection alone?
Source: PLoS One. 2021 Jan 19;16(1):e0245074. doi: 10.1371/journal.pone.0245074 (PMC7815106; doi:10.1371/journal.pone.0245074)
Supplement: S1 Appendix — (PDF) [file pone.0245074.s001.pdf]

Appendix S1. Value orientations and survey items

Orientation	Item
Egoistic	Social power: control over others, dominance
	Wealth: material possessions, money
	Authority: the right to lead or command
	Influential: having an impact on people and events
Altruistic	Equality: equal opportunity for all
	A world at peace: free of war and conflict
	Social justice: correcting injustice, care for the weak
	Helpful: working for the welfare of others
Biospheric	Respecting the earth: harmony with other species
	Preventing pollution: protecting natural resources
	Unity with nature: fitting in with nature
	Protecting the environment: preserving nature
