# Supplementary material for: Public support for restoration: Does including ecosystem services as a goal engage a different set of values and attitudes than biodiversity protection alone?
Source: PLoS One. 2021 Jan 19;16(1):e0245074. doi: 10.1371/journal.pone.0245074 (PMC7815106; doi:10.1371/journal.pone.0245074)
Supplement: S2 Appendix — Asterisk indicates reverse-coded item. (PDF) [file pone.0245074.s002.pdf]

Appendix S2. Attitude axes, scales, and survey items. Asterisk indicates reverse-coded item.

Axis	Scale	Item
Preservation	Enjoyment of nature	I really like going on trips to the countryside, for example, to forests or fields.
	Environmental movement activism	I don't think I would help to raise funds for environmental protection.*
	Environmental threat	The balance of nature is very delicate and easily upset.
	Ecocentric concern	Nature is valuable for its own sake.
Utilization	Altering nature	When nature is uncomfortable and inconvenient for humans, we have every right to change and remake it to suit ourselves.
	Human dominance over nature	Humans were created, or evolved, to dominate the rest of nature.
	Human utilization of nature	Protecting people's jobs is more important than protecting the environment.
	Conservation motivated by anthropocentric concern	Conservation is important, even if it lowers people's standard of living.*
