# Supplementary material for: Public support for restoration: Does including ecosystem services as a goal engage a different set of values and attitudes than biodiversity protection alone?
Source: PLoS One. 2021 Jan 19;16(1):e0245074. doi: 10.1371/journal.pone.0245074 (PMC7815106; doi:10.1371/journal.pone.0245074)
Supplement: S3 Appendix — (PDF) [file pone.0245074.s003.pdf]

Appendix S3. Survey questions analyzed for this paper

Introduction to both scenarios:

All over Australia, projects are underway or planned to restore degraded lands. This may involve tree planting, fighting weeds, reintroducing native animals that have become scarce, improving waterways, and many other kinds of actions.

[Photographs of people engaging in restoration activities]

Restoration projects in Australia have different kinds of goals. For instance, a project might be aimed at connecting up patches of forest, so that animals can move between them. Or it might be aimed at preventing soils from eroding and clogging up streams. Or it might be aimed at providing a green space for people to take bushwalks.

[Photographs of people walking in nature and a kangaroo hopping in the wild]

Restoration projects can yield different benefits to humans and to society. In this survey, we are interested in what you think about these benefits. Some might affect you personally; others might benefit society more generally; or you might not think that some results of the restoration are beneficial at all. We want your honest opinion.

On the next page, you'll start answering questions about two types of restoration projects: one that is general to all of Australia, and another that is specific to the region you live in.

Biodiversity Only Scenario (BO):

This scenario concerns a tiny island, offshore of mainland Australia, that is home to colonies of seabirds, including gulls, albatrosses, and penguins. No people live there, and no hunting or fishing are permitted. Because of the difficulty making a landing on the island, the only human visitors it receives are scientists who monitor the bird populations there.

[Photographs of rats and seabirds]

Many years ago, rats were accidentally introduced to the island. Rats eat seabird eggs and also kill chicks. Because the rats are not native to the island, some of the birds have no natural defenses against them, and are now threatened with extinction.

A nonprofit organisation has proposed to trap and remove all the rats from the island. However, government funding will not pay the entire cost. If sufficient private donations are not received by the nonprofit, the rat removal program will not be implemented.

Would you be willing to make a one-time donation from your private income to the not-for-profit organisation, so that the island can be made free of rats for the benefit of seabirds?

If yes:

What amount is closest to what you would be willing to pay, as a one-time donation, so that the site can be restored to benefit seabirds? Remember that the amount you donate will reduce the money you have to spend on other things.

$1	$5	$20	$65	$225	don't know
$2	$8	$30	$100	$350	more than $500
$3	$12	$45	$150	$500	

If no:

What is your MAIN reason for choosing not to donate?

- I don't think the benefits are worth paying for.
- I think there are downsides to restoration that outweigh the benefits.
- I am not confident that the restoration project would actually provide the benefits.
- I think government money should support the whole project, without need for private donations.
- I don't give to charitable or not-for-profit organisations.
- I don't trust that my money would actually go to the restoration project.
- Other (please specify in box below)

Biodiversity plus Ecosystem Services scenario (BES):
Regional variant example: Victoria-Gippsland & Southeast

Next, we'd like to show you a restoration scenario for your local region. Please indicate the part of the state in which you live:

- VIC - Greater Melbourne region
- VIC - Barwon and Southwest
- VIC - Gippsland and Southeast
- VIC - Hume and Northeast
- VIC - Grampians
- VIC - Loddon Mallee

This restoration site measures 152 hectares (1 hectare is about the size of a rugby field). The site was formerly box gum grassy woodland, dominated by yellow box, white box, and red gum trees. Woodland birds like the regent honeyeater frequent these trees, squirrel gliders are common in their canopy, and the groundcover includes grasses, tiny orchids, and ferns.

[Pictures of habitat and local wildlife mentioned in scenario]

Due to various environmental changes and land-use decisions, the bush at the restoration site is now in a degraded state. Depending on how the site is replanted and managed into the future, it may be possible to bring about several different benefits, such as habitat for the spotted-tail quoll (a threatened species); recreational access; carbon storage; harvests of timber products; benefits to nearby farms; increases to water supply; and water pollution prevention, among others—but not all at the same time. In other words, there are tradeoffs in how the site is restored and managed—if some benefits are prioritised, others might not occur, or there may even be some negative impacts.

Go to the next page to begin answering questions about this project. You won't be able to return to this page later, so if you want to refer back to this project description again, <u>click here to save it as a pdf file.</u>

Biodiversity plus Ecosystem Services scenario (BES):
Regional variant example: Western Australia and Perth

We'd like to show you a restoration scenario for your local region. Please indicate the part of the state in which you live:

- ○ **WA - Perth area**
- ○ **WA - Southwest and Great Southern**
- ○ **WA - Goldfields and Esperance**
- ○ **WA - Gascoyne and Midwest**
- ○ **WA - Wheatbelt**
- ○ **WA - Kimberley and Pilbara**

This restoration site measures 152 hectares (1 hectare is about the size of a rugby field). The site was formerly a marri woodland of the type that used to be very common on the Swan coastal plain, known for both marri trees and grass trees, or yakka gums, in the understory. This type of open, grassy bush is good habitat for kangaroos and for a wide variety of spring wildflowers.

[Pictures of habitat and local wildlife mentioned in scenario]

Due to various environmental changes and land-use decisions, the bush at the restoration site is now in a degraded state. Depending on how the site is replanted and managed into the future, it may be possible to bring about several different benefits, such as habitat for the numbat (a threatened species); recreational access; carbon storage; harvests of timber products; benefits to nearby farms; increases to water supply; and water pollution prevention, among others—but not all at the same time. In other words, there are tradeoffs in how the site is restored and managed—if some benefits are prioritised, others might not occur, or there may even be some negative impacts.

Go to the next page to start answering questions about this project. You won't be able to come back to this page later, so if you want to refer to the project description again, click here to save it as a pdf file.

Preferred benefit from BES Scenario:

This is a list of all the benefits that could come from the restoration project you read about.

Which ONE of these benefits do you prefer MOST?

- ○ **public access for recreation**
- ○ **jobs, training, or environmental education**
- ○ **improvements to water quality**
- ○ **increases to amount of water supply**
- ○ **soil improvements**
- ○ **farm benefits**
- ○ **harvest of forest products**
- ○ **bigger or better habitat for native plants and animals**
- ○ **protection for threatened species**
- ○ **carbon storage**

Dynamic response- WTP question

You chose **"bigger or better habitat for native plants and animals"**. Suppose that the local not-for-profit organisation performing the restoration project plans to focus on bigger or better habitat for native plants and animals.

Some of the funding for the restoration project will come from government grants, but the rest will have to come from donations by the public. If not enough donations are received, the project will not be implemented.

Would you be willing to make a one-time donation from your private income to the not-for-profit organisation, so that the site could be restored and managed to prioritise bigger or better habitat for native plants and animals?

Yes, I would be willing to donate some money.

No, I would not be willing to donate any money.

If yes:

What amount is closest to what you would be willing to pay, as a one-time donation, so that the site could be restored to provide your preferred benefit? Remember that the amount you donate will reduce the money you have to spend on other things.

$1	$5	$20	$65	$225	don't know
$2	$8	$30	$100	$350	more than $500
$3	$12	$45	$150	$500	

If no:

What is your MAIN reason for choosing not to donate?

- ○ I don't think the benefits are worth paying for.
- ○ I think there are downsides to restoration that outweigh the benefits.
- ○ I am not confident that the restoration project would actually provide the benefits.
- ○ I think government money should support the whole project, without need for private donations.
- ○ I don't give to charitable or not-for-profit organisations.
- ○ I don't trust that my money would actually go to the restoration project.
- ○ Other (please specify in box below)

Benefits to nature vs. benefits to people in BES Scenario

Thinking back to the *local* restoration project you read about, how much do you agree with the following statements?

	Strongly Agree	Agree	Neither Agree nor Disagree	Disagree	Strongly Disagree
I expect the local restoration project would have important benefits to people.	○	○	○	○	○
I expect the local restoration project would have important benefits to nature.	○	○	○	○	○
I expect that I or my family would personally benefit from the local restoration project.	○	○	○	○	○

Perception of disservices

Some people are concerned about potential downsides to restoration projects. Below are some arguments that people have made about different projects elsewhere in Australia. How strongly do you agree or disagree with these arguments, in general?

	Strongly Agree	Agree	Neither Agree nor Disagree	Disagree	Strongly Disagree
Restored forests will get overgrown and cause bushfires.	○	○	○	○	○
Restored forests will take away farmland and hurt the economy.	○	○	○	○	○
Restored forests will be uglier than the scenic countryside we have now.	○	○	○	○	○
Restored forests will use up too much groundwater.	○	○	○	○	○
Restored forests will attract pests.	○	○	○	○	○
